# Combined application of pharamcokinetic DCE‐MRI and IVIM‐DWI could improve detection efficiency in early diagnosis of ductal carcinoma in situ

**DOI:** 10.1002/acm2.12624

**Published:** 2019-05-23

**Authors:** Wei‐jing Tao, Hui‐xin Zhang, Lian‐mei Zhang, Feng Gao, Wei Huang, Yan Liu, Yan Zhu, Gen‐ji Bai

**Affiliations:** ^1^ Department of Nuclear Medicine The Affiliated Huai'an No. 1 People's Hospital of Nanjing Medical University Huai’an City Jiangsu Province China; ^2^ Department of Ultrasound The Affiliated Huai'an No. 1 People's Hospital of Nanjing Medical University Huai’an City Jiangsu Province China; ^3^ Department of Pathology The Affiliated Huai'an No. 1 People's Hospital of Nanjing Medical University Huai’an Jiangsu China; ^4^ Department of Medical Imaging Jinling Hospital, Medical School of Nanjing University Nanjing City Jiangsu Province China; ^5^ Department of Radiology The Affiliated Huai'an No. 1 People's Hospital of Nanjing Medical University Huai’an City Jiangsu Province China

**Keywords:** diffusion‐weighted imaging, ductal carcinoma in situ, dynamic contrast‐enhanced magnetic resonance imaging, intravoxel incoherent motion

## Abstract

**Purpose:**

Ductal carcinoma in situ (DCIS) is a precursor of invasive ductal breast carcinoma (IDC). This study aimed to use pharamcokinetic dynamic contrast‐enhanced magnetic resonance imaging (DCE‐MRI) and intravoxel incoherent motion diffusion‐weighted imaging (IVIM‐DWI) for the early diagnosis of DCIS.

**Methods:**

Forty‐seven patients, including 25 with DCIS (age: 28–70 yr, mean age: 48.7 yr) and 22 with benign disease (age: 25–67 yr, mean age: 43.1 yr) confirmed by pathology, underwent pharamcokinetic DCE‐MRI and IVIM‐DWI in this study. The quantitative parameters *K^trans^*, *K_ep_*, *V_e_*, *V_p_*, and *D*, *f*, D* were obtained by processing of DCE‐MRI and IVIM‐DWI images with Omni‐Kinetics and MITK‐Diffusion softwares, respectively. Parameters were analyzed statistically using GraphPad Prism and MedCalc softwares.

**Results:**

All low‐grade DCIS lesions demonstrated mass enhancement with clear boundaries, while most middle‐grade and high‐grade DCIS lesions showed non‐mass‐like enhancement (NMLE). DCIS lesions were significantly different from benign lesions in terms of *K^trans^*, *K_ep_*, and *D* (*t* = 5.959, *P* < 0.0001; *t* = 5.679, *P* < 0.0001; and *t* = 5.629, *P* < 0.0001, respectively). The AUC of *K^trans^*, *K_ep_*, *D* and the combined indicator of *K^trans^*, *K_ep,_* and *D* were 0.936, 0.902, 0.860, and 0.976, respectively. There was a significant difference in diagnostic efficacy only between *D* and the combined indicator (*Z* = 2.408, *P* = 0.016).

**Conclusion:**

DCE‐MRI and IVIM‐DWI could make for the early diagnosis of DCIS, and reduce the misdiagnosis of DCIS and over‐treatment of benign lesions.

## 1 INTRODUCTION

Breast cancer, the most common female cancer, is highly heterogeneous and is probably caused by numerous changes in the genome of specific cells over extended time periods.^1^, ^2^ Changes of normal‐phenotype breast cells to cancerous‐phenotype cells are affected by a series of factors, such as the local and non‐native environment, lifestyle, dietary habits, and genetic inheritance, which can disrupt cells' physical characteristics, behavior, and communication pathways.^3^ Ductal carcinoma in situ (DCIS) of the breast, a noninvasive and nonobligate precursor lesion, represents a transition from normal tissue to an invasive ductal breast carcinoma (IDC) through a multifactorial process.^2^, ^4^

According to the World Health Organization classification of breast tumors in 2012,^5^ DCIS lesions could be classified as high‐grade, middle‐grade, and low‐grade tumors, which have varying prognoses due to distinct molecular markers and genetic signatures.^6^ High‐grade DCIS seems to progress to IDC more rapidly and more frequently than low‐grade DCIS,^7^, ^8^, ^9^ whereas low‐grade DCIS could be more indolent to change or might progress to certain well‐differentiated types of cancer.^10^ However, DCIS requires surgical locoregional treatment to avoid recurrence or progression to IDC.^11^, ^12^, ^13^ Therefore, it is necessary to diagnose DCIS early by noninvasive examinational methods, especially high‐grade DCIS.

Preoperatively diagnosed DCIS accounted for only 2% of breast cancers in 1980, but the proportion increased to approximately 20% in 2002 with mammographic imaging techniques.^14^, ^15^ Microcalcification, as the main feature of DCIS, is more likely to be detected by mammography than other imaging methods, such as MR.^10^, ^16^ However, only 50%–75% of DCIS lesions show microcalcification,^17^ which are frequently misdiagnosed on mammographs, with a sensitivity of 27%–80%.^18^

Recently, with the application of the Breast Imaging Reporting and Data System (BI‐RADS), MRI has become a powerful imaging method for diagnosis of DCIS lesions, with a sensitivity of 96% and the negative‐predictive value (NPV) is 98.24%. However, the specificity and positive‐predictive value (PPV) of MRI in DCIS are 75.67% and 57.14%, respectively,^19^ because DCIS is easily confused with benign lesions in terms of morphology and semi‐quantitative features.^20^ Excising benign breast lesions is an overtreatment, according to BI‐RADS. Therefore, it is necessary to distinguish benign lesions from DCIS of the breast on MRI.

Functional MRI has been widely applied in the diagnosis of breast disease. Pharmacokinetic dynamic contrast‐enhanced MRI (DCE‐MRI) is a sensitive technique that reflects physiological characteristics of lesion microvasculature.^21^ DCE‐MRI parameters for quantitative measure of perfusion, including *K^trans^*, *K_ep_*, *V_e_,* and* V_p_*, reflect tumor angiogenesis density, vascular permeability, and tumor neoangiogenesis blood flow.^22^, ^23^ Additionally, diffusion‐weighted imaging (DWI), another noninvasive quantitative MRI method, could reflect tumor cytoarchitecture and distinguish pseudo‐random movements.^24^ Based on the theory of intravoxel incoherent motion (IVIM), the new DWI analysis model can be performed with >2 b‐values with the following parameters: true diffusion coefficient *D*, the pseudo‐diffusion coefficient D*, and the perfusion fraction *f*.^10^

However, DCE‐MRI and IVIM‐DWI are rarely used to distinguish DCIS from benign lesions of the breast. Herein, we used these techniques to diagnose DCIS to identify DCIS with higher specificity.

## 2 MATERIALS AND METHODS

### 2.1 Study population

This study was conducted in accordance with the standards of the local ethics committee and obtained informed consent from all individual participants. Patients treated between September 2016 and April 2018 were collected based on the following three criteria: (a) breast examination was performed using DCE‐MRI and IVIM‐DWI before treatment and puncture; (b) all lesions were classified as BI‐RADS level 4 and had similar enhanced shapes and time‐signal intensity curve (TIC) types in conventional MRI scans; and (c) every lesion was proven by pathology to be DCIS or benign.

Forty‐seven patients met the above requirements, including 25 cases of DCIS (28–70 yr old, mean age: 48.7 yr old) and 22 cases of benign disease (25–67 yr old, mean age: 43.1 yr old). The DCIS group had three cases of low‐grade DCIS lesions, 16 cases of middle‐grade DCIS lesions, and six cases of high‐grade DCIS lesions. The benign lesions group had seven cases of fibroadenoma, six cases of adenosis, three cases of intraductal papilloma, two cases of mammary ductal dysplasia, and five cases of inflammation.

### 2.2 MRI protocol

MRI was performed with the patient lying in the prone position, by using a four‐channel bilateral breast coil that covered both breasts, with a 3.0 T MRI scanner (Verio; Siemens Healthcare, Erlangen, Germany). All patients were examined using DCE‐MRI and IVIM‐DWI sequences. The parameters of the sequence were described as following: DCE‐MRI: (a) Axial, vibe fat‐suppressed (FS) T1‐weighted imaging (T1WI) sequences with repetition time/echo time (TR/TE) of 3.61/0.96 ms; flip angle of 3°, 6°, 9°, 12°, and 15°, successively; 30 slices; a field of view [FOV] of 380–420 mm; a matrix size of 272 × 320; slice thickness of 4 mm; and acquisition time of 8 s per scan. (b) After the above five sequences, a similar sequence with a flip angle of 12° was performed for 40 scans, continuously, and the MRI contrast agent was injected into vein at the end of the second scan. (c) Patients were injected with 0.2 ml/kg gadodiamide (General Pharmaceutical Co., Shanghai, China) in an antecubital vein, via a catheter, using a power injector (Medrad, Warrendale, PA) at a speed of 2 ml/s, followed by a saline flush (20 ml) at 2 ml/s.

IVIM: The axial, echo planar sequence had a TR/TE of 6500 /91 ms; a slice thickness of 4 mm, FOV of 380 × 260 mm; 24 slices; b‐values were 0, 50, 100, 150, 200, 400, 600, and 1000; and the scanning time was 7 min and 29 s.

### 2.3 Image postprocessing

All images were transferred in Communications in Medicine (DICOM) format. The DCE‐MRI and IVIM‐DWI images were postprocessed by Omni‐Kinetics software (Version 2.06, General Pharmaceutical Co., Shanghai, China) and MITK‐Diffusion software (2014.10.02, German Cancer Research Center, Heidelberg, Germany), respectively.

The modified Tofts model was used in postprocessing of DCE‐MRI, with the following equations:^25^

$$\begin{array}{c} C_{t}\left(t\right)=V_{p}C_{p}\left(t\right)+K^{\mathrm{trans}}\int_{0}^{t}C_{p}\left(t^{\prime }\right)exp\left(\frac{-K^{\mathrm{trans}}}{V_{e}}\right)\left(t-t^{\prime }\right)dt^{\prime }\\ K_{\mathrm{ep}}=\frac{K^{\mathrm{trans}}}{V_{e}} \end{array}$$

where *C_t_(t)* is the concentration of the agent in the voxel at time *t*, while *C_p_* is the concentration of the agent in the plasma volume. *V_e_* is the proportional volume of the extravascular extracellular distribution space (EES). *K^trans^* is the volume transfer constant between the plasma and EES. *V_p_* is the proportional blood plasma volume. *K_ep_* is the diffusion rate constant EES to plasma.

The following biexponential (IVIM) equation was used:

$$S_{b}/S_{0}=fe^{-bD*}+{\left(1-f\right)}^{-bD}$$

where *S_b_* is the mean signal intensity, *S*_0_ is the signal reference, *b* stands for the* b*‐value, and *f* is the fraction of perfusion. D* is the diffusion of the perfusing fraction and *D* is the diffusion of the nonperfusing fraction.

The *K^trans^*, *K_ep_*, *V_e_*, and *V_p_* maps were obtained by postprocessing of DCE‐MRI images with Omni‐Kinetics software. The *D*, *f*, and D*maps, as IVIM‐DWI parameters, were generated by MITK‐Diffusion software postprocessing.

### 2.4 Statistical analysis

Statistical analyses were performed by GraphPad Prism version 6.0 and MedCalc version 15.0. A parametric test (unpaired *t*‐test) was applied when normality assumptions and homogeneity of variance were satisfied. Otherwise, the equivalent non‐parametric test (Mann–Whitney U test) was used. *P* < 0.05 indicated statistical significance.

Receiver operating characteristic (ROC) curves were drawn and the accuracy of parameters that yielded significance differences was selected by using the area under the ROC curve (AUC). *Z*‐tests were performed with the ROC of each parameter by using MedCalc statistical software, and the diagnostic value of each parameter was compared.

## 3 RESULTS

### 3.1 Lesion morphological features

**Figure 1:**
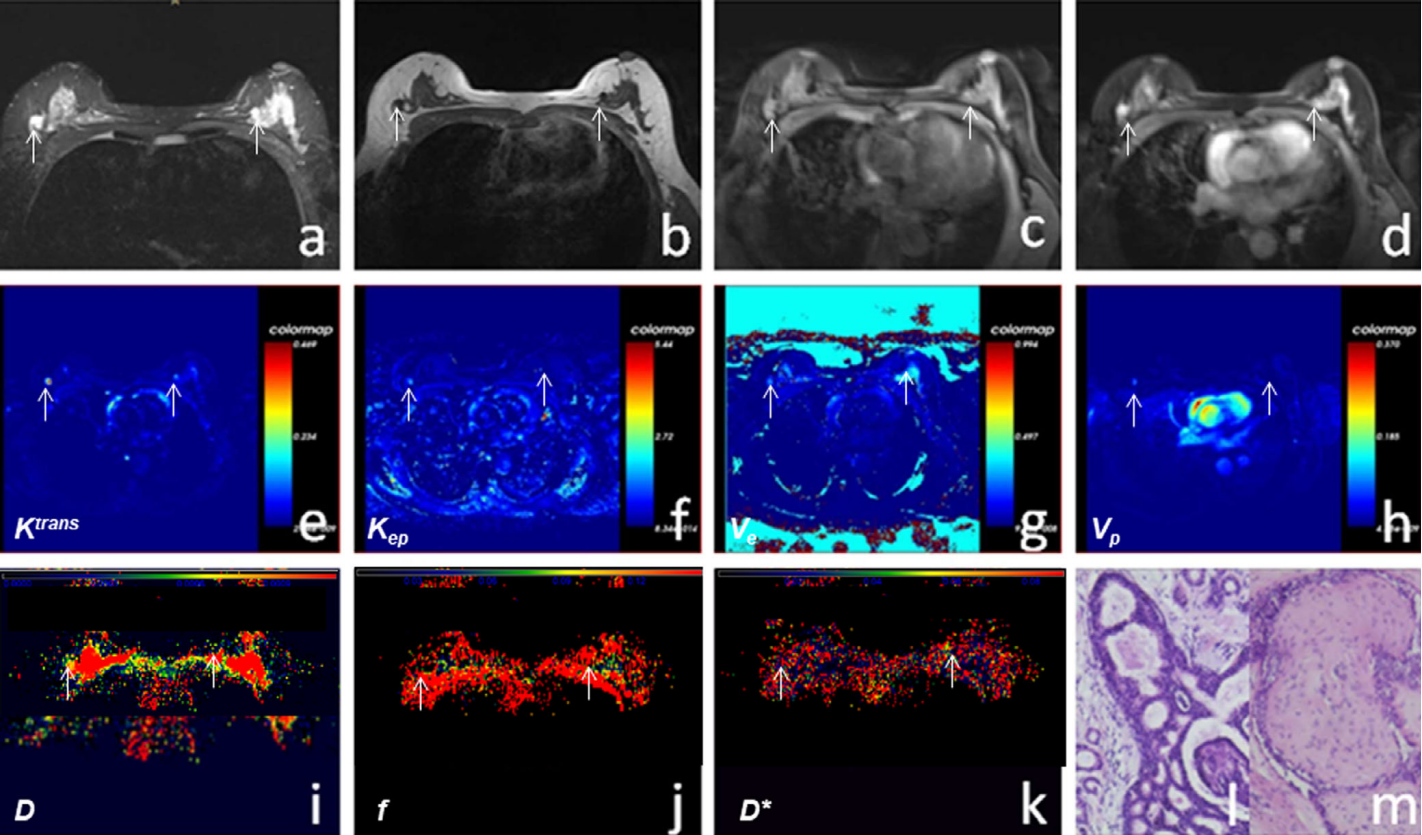
A 49‐year‐old woman had bilateral breast tumors for >7 months, without pain and fever. (a–c) T2WI, T1WI, and T1WI‐FS scans showed bilateral breast lesions with hypointensity on T1WI and hyperintensity on T2WI. (d) Enhanced T1WI‐FS showed bilateral breast lesions that were significantly enhanced, in mass enhancement. (e–h) Bilateral breast tumors are shown in* K^trans^*, *K_ep_*, *V_e_*, and *V_p_* maps, respectively, by postprocessing of dynamic contrast‐enhanced magnetic resonance imaging. (i–k) The *D*, *f,* and D* maps were obtained, respectively, by postprocessing of intravoxel incoherent motion diffusion‐weighted imaging images. (l–m) Hematoxylin and eosin‐stained (×200) images showed that the left lesion was a fibroadenoma and the right lesion was ductal carcinoma in situ.

**Figure 2:**
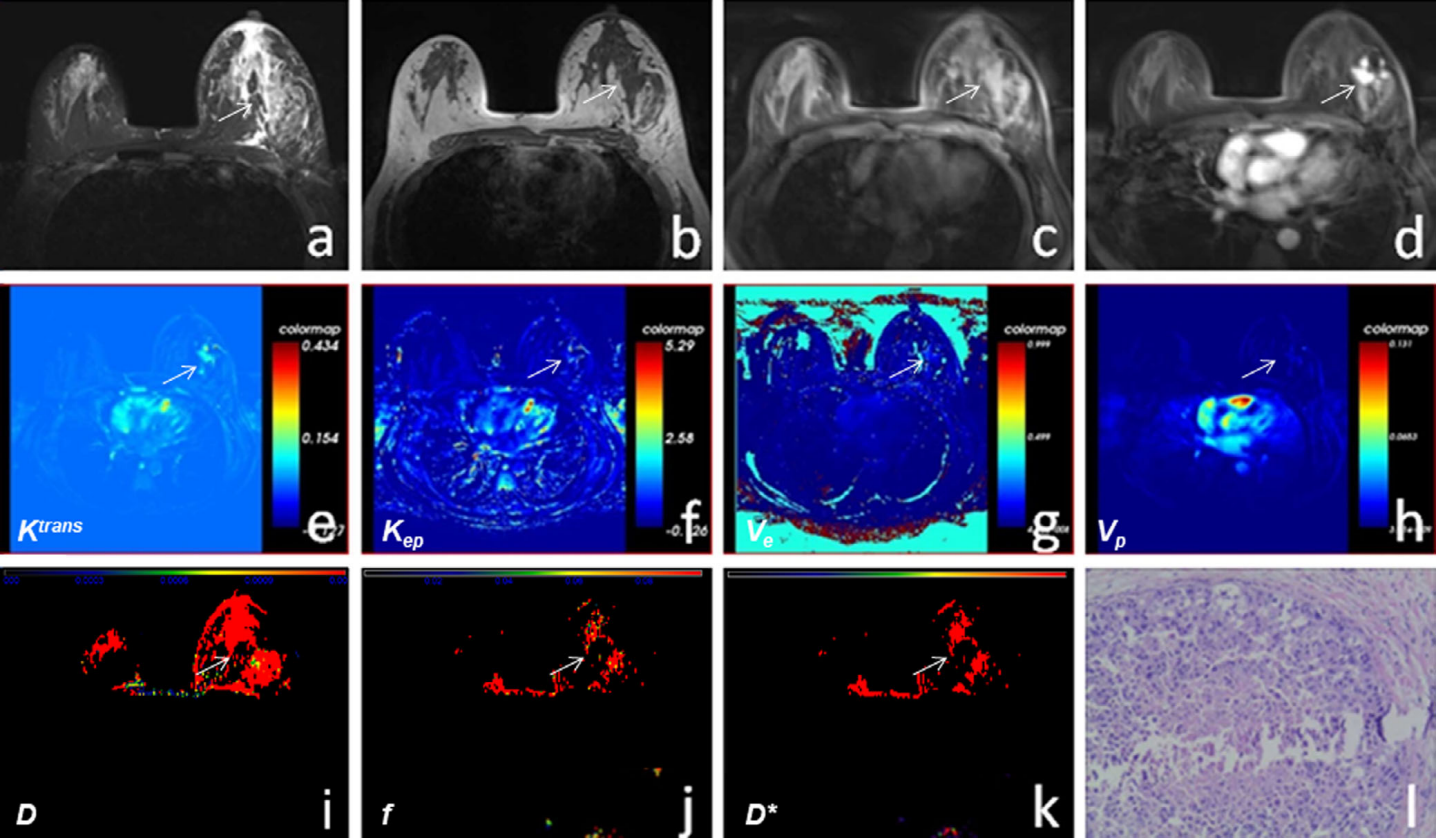
A 51‐yr‐old female exhibited a left breast mass for 1 month. (a–c) T2WI, T1WI, and T1WI‐FS scans showed the left breast lesion was hypointense on T1WI and hyperintense on T2WI without boundaries. (d) Enhanced T1WI‐FS showed that the left breast lesion exhibited non‐mass‐like enhancement. (e–h) The* K^trans^*, *K_ep_*, *V_e_*, and *V_p_* maps with the left breast lesion were obtained respectively by postprocessing of dynamic contrast‐enhanced magnetic resonance imaging. (i–k) The *D*, *f,* and D* maps were obtained by postprocessing of intravoxel incoherent motion diffusion‐weighted imaging images. (l) Hematoxylin and eosin‐stained (×200) images showed that the left lesion was ductal carcinoma in situ.

**Figure 3:**
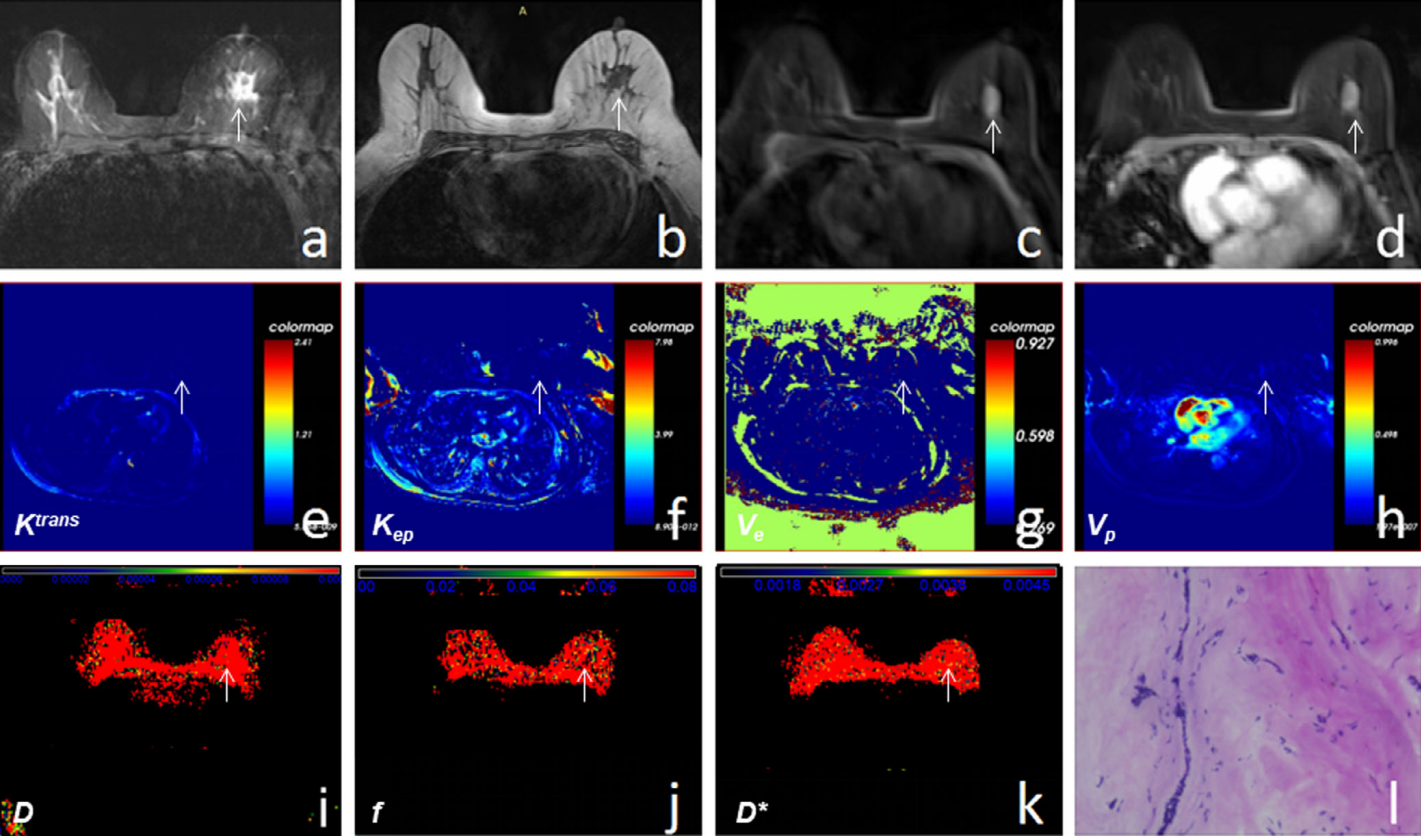
A 61‐yr‐old woman reported a left breast nodule for 5 days. (a–c) T2WI, T1WI, and T1WI‐FS scans showed the left breast lesion was hypointense on T1WI and hyperintense on T2WI. (d) Enhanced T1WI‐FS showed that the left breast lesion exhibited mass enhancement. (e–h) The* K^trans^*, *K_ep_*, *V_e_*, and *V_p_* maps obtained for the left breast lesion, by postprocessing of dynamic contrast‐enhanced magnetic resonance imaging. (i–k) The *D*, *f*, and D* maps were obtained by postprocessing of intravoxel incoherent motion diffusion‐weighted imaging images. (l**)** Hematoxylin and eosin‐stained (×200) image showed that the left lesion was fibroadenoma.

**Figure 4:**
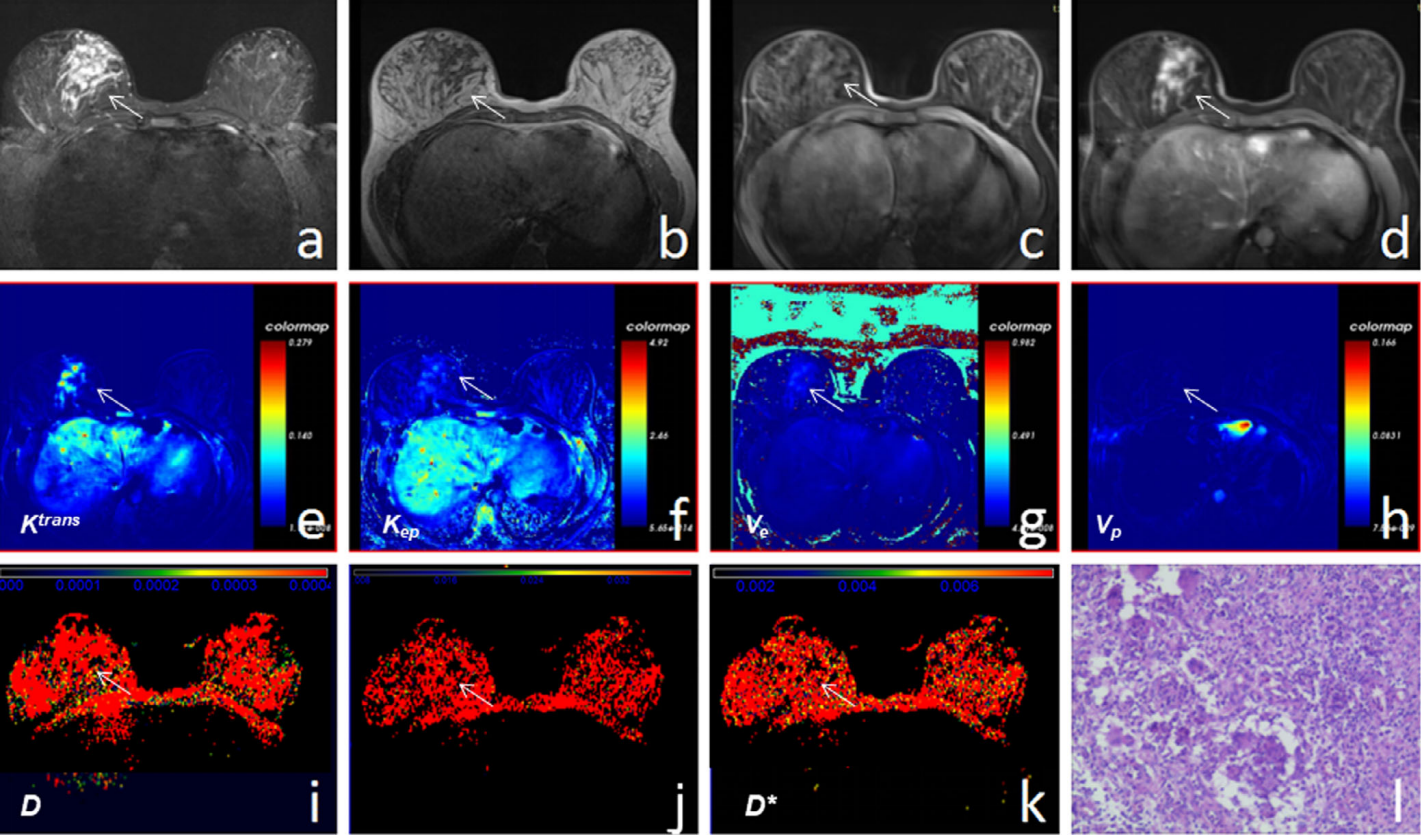
A 26‐yr‐old female reported a right breast lesion for 2 months that gradually enlarged, without pain and fever. (a–c) T2WI, T1WI, and T1WI‐FS scans showed that the left breast lesion was hypointense on T1WI and hyperintense on T2WI without boundaries. (d) Enhanced T1WI‐FS showed that the right breast lesion exhibited non‐mass‐like enhancement. (e–h) The *K^trans^*, *K_ep_*, *V_e_*, and *V_p_* maps obtained for the right breast lesion, by postprocessing of dynamic contrast‐enhanced magnetic resonance imaging. (i–k) The *D*, *f,* and D* maps were obtained by postprocessing of intravoxel incoherent motion diffusion‐weighted imaging images. (l) Hematoxylin and eosin (×200) image showed that the right lesion was inflammatory.

All low‐grade DCIS lesions demonstrated mass enhancement with clear boundaries (3/3) [Fig. 1(d)], while most middle‐grade and high‐grade DCIS lesions showed non‐mass‐like enhancement (NMLE) (16/22) [Table 1, Fig. 2(d)]. Among the benign lesions, all fibroadenomas showed mass enhancement [Figs. 1(d), 3(d)]. In contrast, all inflammatory lesions showed NMLE [Fig. 4(d)]. Adenosis, intraductal papilloma, and mammary ductal dysplasia lesions demonstrated mass enhancement or NMLE, with different probabilities.

**Table 1:** The enhanced features of DCIS and benign lesions in DCE‐MRI.

Tumor group	The mass enhancement		The non‐mass‐like enhancement	
	Number	Percentage	Number	Percentage
DCIS				
Low‐grade	3	100%	0	0
Middle‐grade	4	25%	12	75%
High‐grade	2	33%	4	67%
Benign lesions				
Fibroadenoma	7	100%	0	0
Adenosis	1	17%	5	83%
Intraductal papilloma	2	67%	1	33%
Mammary ductal dysplasia	1	50%	1	50%
Inflammation	0	0	5	100%

DCIS, ductal carcinoma in sit; DCE‐MRI, dynamic contrast‐enhanced MRI.

With mass enhancement, most benign lesions had relatively regular shapes and smooth margins, while most DCIS lesions had small lobulated shapes and irregular margins. In the NMLE, regular ring‐enhancement was seen in inflammatory lesions, while a clustered distribution was mostly seen in DCIS.

### 3.2 Data analysis of DCE‐MRI and IVIM‐DWI parameters

#### 3.2.1 Comparison of parameters between DCIS and benign lesions

**Figure 5:**
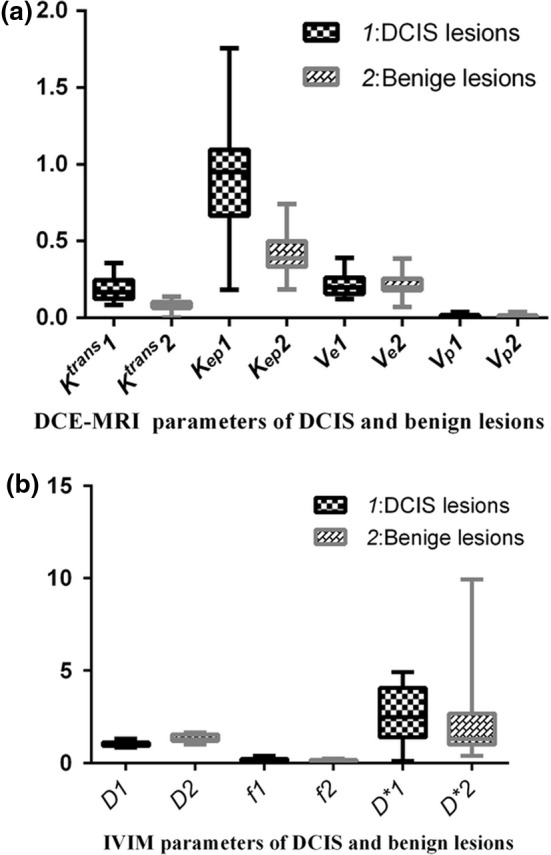
(a) The box and whiskers of dynamic contrast‐enhanced magnetic resonance imaging parameters of ductal carcinoma in situ (DCIS) and benign lesions. (b) The box and whiskers of intravoxel incoherent motion diffusion‐weighted imaging parameters of ductal carcinoma in situ (DCIS) and benign lesions.

Values of *K^trans^* and *K_ep_* (in DCE‐MRI) and *D* (in IVIM) were significantly different between DCIS and benign lesions (*t* = 5.959, *P* < 0.0001; *t* = 5.679, *P* < 0.0001; *t* = 5.629, *P* < 0.0001; Table 2). DCIS lesions had significantly higher *K^trans^* and *K_ep_* values and slightly lower *D* values than benign lesions [Fig. 5(a‐b)].

**Table 2:** DCE‐MRI and IVIM parameters of DCIS and benign lesions.

Parameters	DCIS	Benign lesions	U*/t*	*P*
*K^trans^* (min^−1^)	0.191 ± 0.0815	0.084 ± 0.038	5.959	＜0.001
*K_ep_* (min^−1^)	0.942 ± 0.397	0.462 ± 0.242	5.679	＜0.001
*V*_*e*_	0.225 ± 0.0828	0.210 ± 0.063	266.0	0.853
*V*_*p*_(×10^−3^)	1.464 (3.862, 14.330)	3.500 (3.220, 12.550)	245.0	0.529
*D*(×10^−3^)(mm^2^/s)	1.037 ± 0.139	1.328 ± 0.212	5.629	＜0.001
*f* (%)	16.560 ± 9.574	12.480 ± 6.080	1.717	0.093
D*(×10^−3^)(mm^2^/s)	2.632 ± 1.513	1.307 (1.249, 3.289)	206.5	0.147

DCE‐MRI, dynamic contrast‐enhanced MRI; IVIM, intravoxel incoherent motion; DCIS, ductal carcinoma in situ; *K^trans^*, *K_ep_*, *V*_*e*_, and *V*_*p*_ were parameters of DCE‐MRI; *D*, *f,* and *D* * were parameters of IVIM.

#### 3.2.2 Comparison of parameters among different DCIS grades

No obvious differences in *K^trans^*, *K_ep_*, *V_e_*, and *V_p_* (in DCE‐MRI), and *D*, *f*, and D* (in IVIM) values of lesions were found, indicating different DCIS grades (Table 3).

**Table 3:** DCE‐MRI and IVIM parameters of different grades of DCIS.

Parameters	Low‐grade DCIS	Middle‐grade DCIS	High‐grade DCIS	K	*P*
*K^trans^* (min^−1^)	0.116 (0.086, 0.125)	0.197 ± 0.079	0.230 (0.132, 0.312)	5.671	0.059
*K_ep_* (min^−1^)	0.413 (0.402, 0.852)	0.967 ± 0.421	1.063 (0.808, 1.299)	4.281	0.118
*V*_*e*_	0.195 (0.142, 0.202)	0.240 ± 0.088	0.212 (0.142, 0.286)	1.231	0.540
*V*_*p*_(×10^−3^)	0.003 (0.000, 0.038)	0.010 ± 0.014	0.005 (0.000, 0.016)	0.137	0.934
*D*(×10^−3^)(mm^2^/s)	1.226 (1.091, 1.316)	1.034 ± 0.124	0.907 (0.876, 1.110)	5.275	0.072
*f* (%)	0.225 (0.218, 0.271)	0.156 ± 0.099	0.117 (0.036, 0.221)	4.022	0.134
D*(×10^−3^)(mm^2^/s)	2.183 (0.123, 3.127)	2.756 ± 1.510	1.826 (0.779, 3.868)	1.536	0.464

DCE‐MRI, dynamic contrast‐enhanced MRI; IVIM, intravoxel incoherent motion; DCIS: ductal carcinoma in situ; *K^trans^*, *K_ep_*, *V*_e_, and *V*_p_ were parameters of DCE‐MRI; *D*, *f,* and *D* * were parameters of IVIM.

#### 3.2.3 Diagnostic efficiency test of parameters

**Figure 6:**
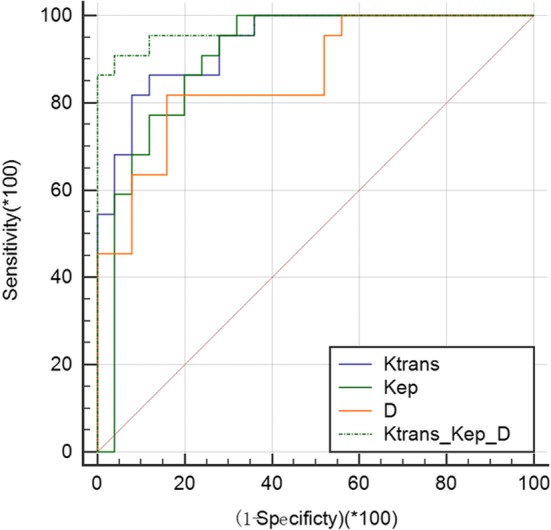
Receiver operating characteristic curves of the parameters *K^trans^*, *K_ep_*, *D*, and the combined indicator of *K^trans^*, *K_ep_*, and *D* to diagnosis DCIS and benign lesions.

ROC curves were used to evaluate the diagnostic efficiency of parameters between DCIS and benign lesions [Table 4, Fig. 6]. All AUCs of* K^trans^*, *K_ep_*, and *D* exceeded 85%, and all of the sensitivity and specificity values were >80%. The cutoff value of every diagnostic parameter calculated on generating ROC curves is listed in Table 4. An indicator combining *K^trans^*, *K_ep_*, and *D* had a higher diagnostic efficiency, with AUC of 0.976, 90.91% sensitivity, and 95% specificity.

**Table 4:** The diagnostic efficiency test of parameters between DCIS and benign lesions.

Parameters	AUC	Cutoff Point	Sensitivity	Specificity
*K^trans^*	0.936	0.114	86.36%	88.46%
*K_ep_*	0.902	0.636	86.36%	80.77%
*D*	0.860	1.177	81.82%	84.00%
*K^trans^* + *K_ep_* + *D*	0.976	/	90.91%	95.00%

DCIS, ductal carcinoma in situ; AUC, area under the curve; *K^trans^* and *K_ep_* were parameters of DCE‐MRI; *D* was one of IVIM parameters.

#### 3.2.4 Comparison of diagnostic efficiency among parameters

There was a statistical difference in AUC only between the parameter *D* and the combined indicator of *K^trans^*, *K_ep_*, and *D* (*Z* = 2.408, *P* = 0.016). And there were no significant differences in diagnostic efficiency among other parameters (Table 5).

**Table 5:** The comparison of diagnostic efficiency among *K^trans^*, *K_ep_*, *D* and *K^trans^* + *K_ep_* + *D*.

Parameters	*K^trans^*		*K_ep_*		*D*	
	*Z*	P	*Z*	P	*Z*	P
*K^trans^*						
*K_ep_*	1.058	0.290				
*D*	1.242	0.214	0.691	0.490		
*K^trans^* +*K_ ep_*+*D*	1.520	0.129	1.767	0.077	2.408	0.016

*K^trans^* and *K_ep_* were parameters of DCE‐MRI; *D* was one of IVIM parameters.

## 4 DISCUSSION

DCIS is defined as a malignant proliferation of ductal cells of the breast that does not invade through the basal membrane. However, some high‐grade DCIS lesions could lead to life‐threatening invasive breast cancers if it is left untreated.^26^ Because DCIS lesions are precancerous rather than malignant lesions, they lack the typical manifestations of invasive ductal carcinoma and then it is difficult to distinguish DCIS from benign lesions in morphology. It is very likely to misdiagnose DCIS lesions as benign lesions during a semiyearly assessment. Therefore it is clinically meaningful to distinguish between DCIS and benign lesions.

Breast MRI is used preoperatively with increasing frequency in women with DCIS, where it has shown high sensitivity for detection, especially in cases of high‐grade lesions.^10^ In our study, most middle‐ and high‐grade DCIS lesions were NMLE, which was consistent with Greenwood's standpoint that most DCIS lesions manifested NMLE in enhanced MRI.^27^ Due to the lack of typical morphological features of malignant lesions in DCIS, and its misinterpretation as benign lesions,^28^ DCIS lesions with NMLE distributions must be distinguished from adenosis, mammary ductal dysplasia, and inflammation during the early background parenchymal enhancement of the breast; DCIS lesions with mass enhancement must be differentiated from fibroadenoma, mammary ductal dysplasia, and intraductal papilloma. DCE‐MRI and IVIM‐DWI are two appropriate functional MRI methods that can be used to distinguish DCIS from benign lesions.

The routine DCE‐MRI sequence, which involved flash three‐dimensional FS T1WI with a period of 90 s, had high spatial resolution that allowed observation of morphological features, but it was difficult to identify DCIS and benign lesions. In our study, the DCE‐MRI sequence, involving vibe FS T1WI with a period of 8 s, had a very high temporal resolution and allowed quantitative kinetic enhancement analysis through pharmacokinetic modeling. Pharmacokinetic models can quantify the contrast agent exchange between the intravascular and interstitial spaces, providing measures of tumor blood flow, microvasculature, and capillary permeability.^29^ Sanders et al.^30^ showed that DCIS lesions were rich in vascular endothelial cells, and their vascular structure had changed. Therefore, a method that provided measures of microvasculature and capillary permeability could help to distinguish DCIS from benign lesions. Pharmacokinetic parameters of DCE‐MRI, such as *K^trans^* and *K_ep_*, could show the capillary permeability and improve differentiation between benign and malignant breast lesions.^31^ Huang et al.^32^ investigated the use of the *K^trans^* value to avoid unnecessary biopsies in suspicious lesions. Our research showed that *K^trans^* and *K_ep_* had high diagnostic efficiency for differentiating DCIS lesions from benign lesions, which was in agreement with results from the previous reports.^29^, ^30^, ^31^

As a novel DWI technique, IVIM‐DWI reveals microscopic biological structures and diffusion of water protons in tissue, without the intravenous contrast agent.^33^ Using a series of multiple *b*‐values, DWI signals could show the perfusion of the capillary network in the low *b*‐value range (*b* < 100–150 s/mm^2^), and the diffusion of water protons in the high *b*‐value range.^34^ In the current study, IVIM‐DWI was an effective tool for diagnosing DCIS and other benign breast lesions by means of parameter *D*. In the biexponential IVIM‐DWI model, *D* value could exclude the influence of cell structure and the microcirculatory perfusion effect.^35^ Furthermore, *D* had a higher diagnostic efficiency than parameters *f* and D*, which was consistent with other literature reports.^35^, ^36^ The cutoff value of *D* was 1.177 × 10^−3^ mm^2^/s, with an AUC of 0.860, sensitivity of 81.82%, and specificity of 84.00%. These values were higher than those reported by Mao et al.^37^, probably because of differences between studied samples.

An indicator that combined* K^trans^*, *K_ep_*, and *D* had a higher diagnostic efficiency than any single parameter. Therefore, combining the DCE‐MRI and IVIM‐DWI methods could increase the accuracy of DCIS diagnosis, which was consistent with the study by Ma in terms of the diagnosis of breast tumors.^38^ Statistical analysis showed that, there was no significant difference in diagnostic efficiency among the combined index,* K^trans^*, *K_ep_*, and *D,* except for the diagnostic efficiencies of the combined index and D. As a DWI method, IVIM‐DWI had some value in diagnosing DCIS, although the ability to identify DCIS and IDS was weaker than that of the combined index.^39^

There were some limitations in our study. The number of cases was relatively small, and the interpretability of the results was limited. The differences in parameters among lower‐grade, high‐grade, and middle‐grade DCIS have not been proven conclusively because of the limited numbers of cases. Despite these limitations, our results suggested that DCE‐MRI and IVIM‐DWI could contribute to the diagnosis of DCIS.

## 5 CONCLUSION

In summary, our study showed significant differences in *K^trans^* and *K_ep_* of DCE‐MRI, and *D* of IVIM between DCIS and benign lesions. DCE‐MRI and IVIM‐DWI were helpful for the early diagnosis of DCIS, which could prevent a missed diagnosis of DCIS, and overtreatment of benign lesions as well.

## CONFLICT OF INTEREST

The authors declare no competing financial interests.

6
